# Age-dependent dopamine transporter dysfunction and Serine129 phospho-α-synuclein overload in G2019S LRRK2 mice

**DOI:** 10.1186/s40478-017-0426-8

**Published:** 2017-03-14

**Authors:** Francesco Longo, Daniela Mercatelli, Salvatore Novello, Ludovico Arcuri, Alberto Brugnoli, Fabrizio Vincenzi, Isabella Russo, Giulia Berti, Omar S. Mabrouk, Robert T. Kennedy, Derya R. Shimshek, Katia Varani, Luigi Bubacco, Elisa Greggio, Michele Morari

**Affiliations:** 1 Department of Medical Sciences, Section of Pharmacology, and National Institute of Neuroscience, University of Ferrara, via Fossato di Mortara 17-19, 44121 Ferrara, Italy; 2 Department of Biology, University of Padova, Via Ugo Bassi 58/B, 35131 Padova, Italy; 3 Departments of Pharmacology and Chemistry, University of Michigan, 930 North University, Ann Arbor, MI 48109 USA; 4 Department of Autoimmunity/Transplantation/Inflammation-Neuroinflammation, Novartis Institutes for BioMedical Research, Novartis Pharma AG, 4002 Basel, Switzerland

**Keywords:** α-synuclein, DAT, G2019S knock-in, LRRK2, Parkinson’s disease, VMAT2

## Abstract

Mutations in the leucine-rich repeat kinase 2 (LRRK2) gene are the most common genetic cause of Parkinson’s disease. Here, we investigated whether the G2019S LRRK2 mutation causes morphological and/or functional changes at nigro-striatal dopamine neurons. Density of striatal dopaminergic terminals, nigral cell counts, tyrosine hydroxylase protein levels as well as exocytotic dopamine release measured in striatal synaptosomes, or striatal extracellular dopamine levels monitored by in vivo microdialysis were similar between ≥12-month-old G2019S knock-in mice and wild-type controls. In vivo striatal dopamine release was insensitive to the LRRK2 inhibitor Nov-LRRK2-11, and was elevated by the membrane dopamine transporter blocker GBR-12783. However, G2019S knock-in mice showed a blunted neurochemical and motor activation response to GBR-12783 compared to wild-type controls. Western blot and dopamine uptake analysis revealed an increase in dopamine transporter levels and activity in the striatum of 12-month-old G2019S KI mice. This phenotype correlated with a reduction in vesicular monoamine transporter 2 levels and an enhancement of vesicular dopamine uptake, which was consistent with greater resistance to reserpine-induced hypolocomotion. These changes were not observed in 3-month-old mice. Finally, Western blot analysis revealed no genotype difference in striatal levels of endogenous α-synuclein or α-synuclein bound to DOPAL (a toxic metabolite of dopamine). However, Serine129-phosphorylated α-synuclein levels were higher in 12-month-old G2019S knock-in mice. Immunohistochemistry confirmed this finding, also showing no genotype difference in 3-month-old mice. We conclude that the G2019S mutation causes progressive dysfunctions of dopamine transporters, along with Serine129-phosphorylated α-synuclein overload, at striatal dopaminergic terminals, which are not associated with dopamine homeostasis dysregulation or neuron loss but might contribute to intrinsic dopaminergic terminal vulnerability. We propose G2019S knock-in mice as a presymptomatic Parkinson’s disease model, useful to investigate the pathogenic interaction among genetics, aging, and internal or environmental factors leading to the disease.

## Introduction

Autosomal-dominant missense mutations in the leucine-rich repeat kinase 2 (*LRRK2*) gene (PARK8, OMIM 609007) cause familial late-onset Parkinson’s disease (PD) [59, 95]. *LRRK2* mutations also occur in 1–2% of sporadic cases [28, 70] and recent genome wide association studies (GWAS) showed that common variations in the *LRRK2* locus increase the risk of disease, pointing to a crucial role of LRRK2 in the pathogenesis of PD. LRRK2-associated PD is clinically and pathologically indistinguishable from the idiopathic form [18], although some differences in motor and non-motor features have been reported [46]. The majority of LRRK2 autoptic cases report progressive degeneration of dopamine (DA) neurons located in the substantia nigra pars compacta (SNpc), and α-synuclein (α-syn)/ubiquitin-positive intraneuronal cytoplasmic inclusions in surviving neurons [31, 33], although a pleomorphic pathology associated with other neurodegenerative diseases has also been observed [95]. Despite the undisputed genetic link between LRRK2 mutations and PD, the pathogenic mechanisms through which LRRK2 mutations affect PD onset and progression remain debated [17, 49]. LRRK2 is a complex multi-domain protein belonging to the ROCO family, characterized by the presence of a GTPase and a serine-threonine kinase domain surrounded by a number of protein-protein interaction domains [16, 51]. The most common LRRK2 pathogenic mutations are represented by Gly2019Ser (G2019S) in the kinase domain, followed by the hotspot mutation Arg1441Cys/Gly/His/Ser (R1441C/G/H/S) in the GTPase domain [19, 73]. The G2019S mutation results in a two to threefold increase in LRRK2 kinase activity, which appears to be crucial for LRRK2-induced neurodegeneration in vitro [26, 90, 91]. More recently, the cellular activity of LRRK2, probed with anti-autophosphorylation antibodies against Serine 1292 [67, 72] and by measuring the phosphorylation of a subset of Rab GTPase which are *bona fide* LRRK2 cellular substrates [76], revealed a homogeneous increase of LRRK2 kinase activity in the presence of pathogenic mutations, which is not limited to the G2019S mutant as it occurs in vitro.

Various LRRK2 rodent models have been generated in the attempt to replicate the dysfunction and/or degeneration of the nigro-striatal dopaminergic pathway in vivo. Unfortunately, these models provided conflicting data. Mice overexpressing human G2019S or R1441C/G mutations through BAC technology did not show overt dopaminergic neurodegeneration [39, 40, 53] but reduced striatal DA content or basal extracellular levels in vivo when compared to non-transgenic wild-type controls [4, 53]. Consistently, the K^+^-evoked DA release was reduced in striatal slices from BAC hG2019S mice [40]. In mice where hG29019S [13, 64] or hR1441C [88] overexpression in SNc was achieved through the CMV/PDGF promoter, an 18–50% reduction in the number of nigral DA cells was observed at old ages (16–21 months). In these mice, no changes of in vivo DA content was observed [64], although the K^+^-evoked DA release from striatal slices was reduced [88]. Conditional expression of hG2019S [41] or hR1441C [83] in SNc also did not cause nigral DA neuron loss; only a mild reduction in the density of TH terminals was observed in 16-month-old mice [41]. In these mice, hG2019S overexpression caused a reduction of DA content and release from striatal slices [41]. Lack of nigro-striatal degeneration [37, 74, 93] or changes in DA content [37, 93] were also confirmed in transgenic rats overexpressing hG2019S or hR1441C mutations. In vitro, a reduction of the K^+^-evoked DA release in BAC overexpressing rats was found [74]. Finally, no overt neurodegeneration [29, 82, 92] or changes in striatal DA content [29, 82] were observed in G2019S or R1441C knock-in (KI) mice, although in vivo microdialysis revealed a 60% reduction in both spontaneous and amphetamine-induced DA release in 12-month-old G2019S KI mice [92].

In a previous longitudinal study, we reported that G2019S KI mice had enhanced motor behavior compared to both WT mice and mice carrying the D1994S kinase-dead mutation [43]. In this follow-up study, we sought to investigate the mechanisms underlying such phenotype, and in particular, whether G2019S LRRK2 is associated with dysregulation of nigro-striatal DA transmission. Indeed, in vivo [93] and in vitro [54] evidence that the G2019S mutation can be associated with increased DA release has been presented. Here, different aspects of striatal DA transmission were evaluated, namely the integrity of the nigro-striatal DA pathway, in vivo and in vitro striatal DA release, expression and function of proteins involved in synaptic load (DA transporter, DAT) or vesicle storage (vesicular monoamine transporter type 2; VMAT2) of DA, and, finally, the levels of endogenous α-syn, and its Serine129 phosphorylated (pSer129 α-syn) or 3,4-dihydroxyphenylacetaldehyde (DOPAL)-bound forms, which are considered markers of synaptic damage.

## Materials and methods

### Animals

Male homozygous LRRK2 G2019S KI mice, backcrossed on a C57Bl/6 J background, were used. Mice were obtained from Novartis Institutes for BioMedical Research, Novartis Pharma AG (Basel, Switzerland) [29], and bred in the vivarium of the University of Ferrara. In behavioral and neurochemical studies, male non-transgenic wild-type (WT) mice were littermates obtained from the heterozygous breeding. Otherwise, WT mice were obtained from homozygous breeding. Mice were kept under regular lighting conditions (12 h light/dark cycle) and given food and water ad libitum. Experimental procedures involving the use of animals were approved by the Ethical Committee of the University of Ferrara and the Italian Ministry of Health (licenses 171/2010-B and 318/2013-B). Adequate measures were taken to minimize animal pain and discomfort.

### Behavioral pharmacology

Three behavioral tests specific for different motor abilities, i.e. the bar, drag and rotarod tests, were used as described [43, 84, 85]. Experimenters were unaware of genotype and treatments. Twelve-month-old mice were acutely administered i.p. with the VMAT2 inhibitor reserpine at the doses of 1 or 2 mg/kg [87], or with the DAT inhibitor GBR-12783 at the dose of 6 mg/kg.

The bar test measures the ability of the animal to respond to an externally imposed static posture. Mice were gently placed on a table and forepaws were placed alternatively on blocks of increasing heights (1.5, 3 and 6 cm). The time (in seconds) that each paw spent on the block (i.e. the immobility time) was recorded (cut-off time of 20 s). Performance was expressed as total time spent on the different blocks. The drag test measures the ability of the animal to balance its body posture with the forelimbs in response to an externally imposed dynamic stimulus (backward dragging) [47]. It gives information regarding the time to initiate and execute (bradykinesia) a movement. Animals were gently lifted from the tail leaving the forepaws on the table, and then dragged backwards at a constant speed (about 20 cm/s) for a fixed distance (100 cm). The number of steps made by each paw was recorded. Five to seven determinations were collected for each animal. Finally, the fixed-speed rotarod test integrates different motor parameters such as motor coordination, gait ability, balance, muscle tone and motivation to run. Mice were tested over a wide range of increasing speeds (0–55 rpm; 5 rpm steps, increased every 180 s) on a rotating rod (diameter of the cylinder 8 cm) and the total time spent on the rod was recorded [84, 85]

### In vivo microdialysis

Two concentric microdialysis probes (1 mm Cuprophane membrane with a 6 kDa cut-off; AgnTho’s, Stockolm, Sweden) were stereotaxically implanted under isoflurane anesthesia in both dorsal striata (coordinates from the bregma: AP +0.6, ML ±2.0, DV −2.0) [62]. Twenty-four hours after implantation, probes were perfused (2.1 μl/min) with a modified Ringer solution (in nM CaCl_2_ 1.2; KCl 2.7; NaCl 148 and MgCl_2_ 0.85) and samples were collected every 20 min [6, 45, 87] after a 6 h wash-out period. Experiments were run at 24 and 48 h after implantation, and treatments were randomized. GBR-12783 and Nov-LRRK2-11 were administered at 20 mg/kg (i.p.) and 10 mg/kg (i.p.), respectively.

### Neurochemical analysis using LC-MS

DA, HVA, DOPAC and 3MT concentrations in dialysates were analyzed using a benzyolation derivatization LC-MS method described by [75]. Briefly, 5 μl dialysate samples were derivatized by adding 2.5 μl of 100 mM sodium tetraborate, 2.5 μl of 2% benzoyl chloride in acetonitrile, and 2.5 μl of a stable ^13^C benzoylated isotope internal standard mixture for improved quantitation. A Thermo Fisher Accela UHPLC (Waltham, MA) system automatically injected 5 μl of the sample onto a Waters (Milford, MA) HSS T3 reverse phase HPLC column (1 mm X 100 mm, 1.8 μm). Mobile phase A consisted of 10 mM ammonium formate and 0.15% formic acid. Mobile phase B was pure acetonitrile. Analytes were detected by a Thermo Fisher TSQ Quantum Ultra triple quadrupole mass spectrometer operating in multiple reaction monitoring (MRM) mode. Run times were approximately 6 min and all analytes could be detected well above quantification limits (data not shown).

### Synaptosomes preparation

Mice were anesthetized and decapitated. Striata from each mouse were homogenized in ice-cold 0.32 M sucrose (pH 7.4) with a Teflon-glass homogenizer and centrifuged at 9,500 g for 10 min at 4 °C. The supernatant was then centrifuged at 10,000 g for 20 min at 4 °C, and the pellet processed for (i) release experiments or (ii) DA uptake assay.

#### Release experiments

The pellet was resuspended in 1.5 ml of pre-oxygenated Krebs solution (in mM: NaCl 118.5, KCl 4.7, CaCl_2_ 1.2, MgSO_4_ 1.2, KH_2_PO_4_ 1.2, NaHCO_3_ 25, glucose 10, ascorbic acid 0.05, disodium EDTA 0.03, pH 7.4) and incubated with 50 nM [^3^H]-DA (specific activity 40 Ci/mmol; Perkin-Elmer, Boston, MA, USA) for 25 min at 36.5 °C [48]. At the end of the incubation, 12 ml of pre-oxygenated Krebs were added, then 1 ml aliquots of the suspension (~0.35 mg protein) were injected into nylon syringe filters maintained at 36.5 °C and superfused (0.4 ml/min) with pre-oxygenated Krebs. Under these superfusion conditions, spontaneous [^3^H]-DA efflux is essentially unaffected by reuptake [48]. Sample collection (every 3 min) was initiated after a 20 min period of filter washout. Radioactivity in the samples and in the filters was measured using a Perkin Elmer Tri Carb 2810 TR scintillation counter.

#### DA uptake assay

The pellet was resuspended in ice-cold uptake buffer (in mM: NaCl 125, KCl 5, MgSO_4_ 1.5, CaCl_2_ 1.2, KH_2_PO_4_ 1.5, glucose 10, HEPES 25, pargyline 0.1, ascorbic acid 0.5, pH 7.4) and incubated for 5 min at 37 °C with 20 nM [^3^H]-DA isotopically diluted with varying concentrations of unlabeled DA to obtain final DA concentrations in the 20–2000 nM range. Non-specific DA uptake was evaluated in the presence of 5 μM GBR-12783. The reaction was terminated by filtering the assay mixture through Whatman GF/B glass fiber filters using a Brandel cell harvester (Brandel Instruments, Unterföhring, Germany). The filter-bound radioactivity was counted using a Perkin Elmer Tri Carb 2810 TR scintillation counter. Specific DA uptake, defined as the difference between DA accumulated in the absence and in the presence of GBR-12783, was expressed as pmol/mg protein/min [94]. Protein concentration was determined using a Bio-Rad method with bovine albumin as standard reference. Kinetic parameters (V_max_ and K_m_) were determined using Prism 5.0 (GraphPad Software Inc., San Diego, CA).

### Western blot analysis

Mice were anesthetized and decapitated. Striata were solubilized and homogenized in lysis buffer (RIPA buffer, protease and phosphatase inhibitor cocktail) and centrifuged at 13,000 rpm for 15 min at 4 °C. Supernatants were collected and total protein levels were quantified using the bicinchoninic acid protein assay kit (Thermo Scientific). Thirty micrograms of protein per sample were separated by SDS-PAGE, transferred onto polyvinyldifluoride membrane and tested for the following primary antibodies: rabbit anti-tyrosine hydroxylase (TH) (Merck Millipore, AB152, 1:1000), rabbit anti-DAT (Sigma Aldrich, D6944, 1:1000), rabbit anti-VMAT2 (Sigma Aldrich, V9014, 1:300), rabbit anti-VMAT2 (Miller Lab, Emory University, 1:1000 [15]), rabbit anti-pSer129 α-syn (Abcam, ab51253, 1:1000), rabbit anti pSer1292 LRRK2 (Abcam, ab203181, 1:300). Appropriate horseradish peroxidase-linked secondary antibodies (Merck Millipore, goat anti-rabbit IgG HRP-conjugate 12–348, 1:4000 or goat anti-rat IgG HRP-conjugate AP136P, 1:5000) were then used and immunoreactive proteins were visualized by enhanced chemiluminescence (ECL) detection kit (Pierce™ BCA Protein Assay Kit, Thermo Scientific or ECL+, GE Healthcare). Images were acquired and quantified using the ChemiDoc MP System and the ImageLab Software (Bio-Rad). Membranes were then stripped and re-probed with rabbit anti-GAPDH antibody (Thermo Scientific, PA1-988, 1:1000), rabbit anti-LRRK2 (Abcam, ab133474, 1:300) or rabbit anti-α-syn antibody (Abcam, ab52168, 1:1000). Data were analyzed by densitometry and the optical density of specific target protein bands was normalized to the corresponding housekeeper protein levels.

DOPAL-bound α-syn was revealed using ABPA resin (Sigma Aldrich, A8530) pulldown [35, 66]. Five-hundred micrograms total protein were incubated with 50 μl of the resin overnight at 4 °C shaking. The resin was then pelleted, the supernatant removed and the resin was washed twice with PBS/acetonitrile and water. Protein was collected from the resin by adding 20 μl Laemmli buffer and processed as described above using the anti-α-syn antibody. The band intensity was quantified by Image J software and the pull down protein was compared with the total lysate.

### Immunohistochemistry

Mice were deeply anesthetized with isoflurane and transcardially perfused with 4% paraformaldehyde in Phosphate Buffer Solution (PBS; 0.1 M, pH 7.4). Brains were removed, transferred to a 30% sucrose solution in PBS for cryoprotection and then stored at −80 °C.

### TH, α-syn and pSer129 α-syn immunohistochemistry

Fifty micrometer free-floating sections of striatum (AP from +1.42 to +0.14 from bregma) and SNc (AP from −3.16 to −3.52 from bregma [62]) were rinsed in PBS incubated for 30 min at room temperature with a blocking solution (PBS + BSA 1:50 + Triton X100 0.3%) and then incubated with a rabbit polyclonal antibody raised against TH (ab112; 1:750 in BSA 1% PBST; Abcam, Cambridge, UK), α-syn (ab52168; 1:250 in BSA 1% PBST; Abcam, Cambridge, UK) or pSer129 α-syn (ab52153; 1:200 in BSA 1% PBST; Abcam, Cambridge, UK) overnight at 4 °C. Sections were then rinsed and incubated with an anti-rabbit HRP-conjugated secondary antibody (ab6721, 1:500 in BSA 1% PBST; Abcam, Cambridge UK) and revealed by a DAB substrate kit (ab64238, Abcam, Cambridge, UK). Sections were mounted on gelatinized slides, dehydrated and coverslipped for further analysis. To quantify the levels of expression of α-syn and pSer129 α-syn, the semi-stereological method described by Bourdenx et al. [7] was employed. This method has been rigorously standardized; in fact all serial striatal slices for each animal were taken, marked, put in the same well and exposed to DAB for 1 min (according to the data sheet of Abcam DAB substrate Kit). After being mounted, slides were scanned and the representative surface of the staining in each SN section was determined using a color threshold, then the area was sampled (probes of 50 × 40 μm, space 150 × 120 μm). The Cavalieri principle was applied to evaluate the representative volume of α-syn or pSer129 α-syn expression for each SN. The SN volume obtained from the α-syn staining was used to calculate the pSer129 α-syn expression as a percentage, thus allowing the comparison between the two different groups.

### Stereology and neuron counting

Stereological analysis was performed counting TH+ neurons (phenotypic marker) and cresyl violet stained cells (structural marker) in SNc, according to an unbiased stereological sampling method based on optical fractionator stereological probe [34]. A Leica DM6000B motorized microscope coupled with the Mercator Pro software (Mercator Digital Imaging System, Explora Nova, La Rochelle, France) was used [2, 5, 7]. Counting was performed on at least 5 consecutive 50 μm thick slices, magnified at 40X.

### VMAT2 activity assay

Mice were anesthetized and decapitated. Whole brains were homogenized in ice-cold buffer (4 mM HEPES, 0.32 M sucrose, pH 7.4) and centrifuged at 1,000 g for 10 min at 4 °C [10]. Supernatants were centrifuged at 20,000 g for 20 min at 4 °C, the resulting pellets were resuspended in 1.6 ml of resuspension buffer (0.32 M sucrose, pH 7.4) and subjected to osmotic shock by 10 up-and-down strokes in 6.4 ml of ice-cold water followed by addition of 1 ml of 250 mM HEPES and 1 M potassium tartrate, pH 7.4, to restore osmolarity. Samples were then centrifuged at 20,000 g for 20 min at 4 °C, and supernatants were centrifuged at 120,000 g for 2 h at 4 °C. Final pellets containing synaptic vesicles were resuspended in assay buffer (100 mM potassium tartrate, 25 mM HEPES, 0.1 mM EDTA, 0.05 mM EGTA, 1.7 mM ascorbate, 2 mM ATP disodium salt, pH 7.4) and incubated for 5 min at 37 °C with 20 nM [^3^H]-DA isotopically diluted with varying concentrations of unlabeled DA. Non-specific DA uptake was evaluated in the presence of 10 μM tetrabenazine. The reaction was terminated by filtering the assay mixture through 0.5% polyethylenamine-soaked Whatman GF/B glass fiber filters using a Brandel cell harvester. The filter-bound radioactivity was counted using a Perkin Elmer Tri Carb 2810 TR scintillation counter.

### Data presentation and statistical analysis

Data are expressed as percentage of baseline (behavioral experiments) or absolute values and are mean ± SEM (standard error of the mean) of n mice. Statistical analysis of drug effect was performed by one-way conventional or repeated measure (RM) analysis of variance (ANOVA) followed by the Newman-Keuls test for multiple comparisons, or by two-way ANOVA followed by Bonferroni test for multiple comparisons. The Student *t*-test, two tailed for unpaired data, was used to compare two groups of data. *P*-values <0.05 were considered to be statistically significant.

### Drugs

GBR-12783 dihydrochloride and reserpine were purchased from Tocris Bioscience (Bristol, UK). Nov-LRRK2-11 was obtained from Novartis Institutes for BioMedical Research, Novartis Pharma AG (Basel, Switzerland).

## Results

### The nigro-striatal DA pathway is intact in G2019S KI mice

**Fig. 1:**
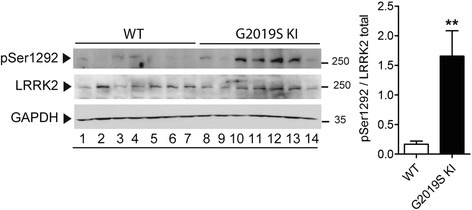
Phosphorylation levels of LRRK2 at Ser1292 (pSer1292) are elevated in G2019S knock-in (KI) mice. Striatal pSer1292 and total LRRK2 levels were measured by Western blotting in 12-month-old G2019S KI mice and age-matched WT controls. Representative blots (left) and quantification (right) are shown. Data are expressed as pSer1292 LRRK2/total LRRK2 and are means ± SEM of 7 animals per group. Statistical analysis was performed with the Student *t*-test, two tailed for unpaired data. **p < 0.01, different from WT

To confirm that the G2019S KI mice under study possess enhanced LRRK2 kinase activity, we monitored LRRK2 autophosphorylation levels at Ser1292 using Western blotting. We found that pSer1292 levels were ~8-fold higher in the striatum of 12-month-old G2019S KI mice compared to age-matched WT littermates (Fig. 1), indicating a clear-cut gain of kinase activity in the presence of the G2019S mutation.

**Fig. 2:**
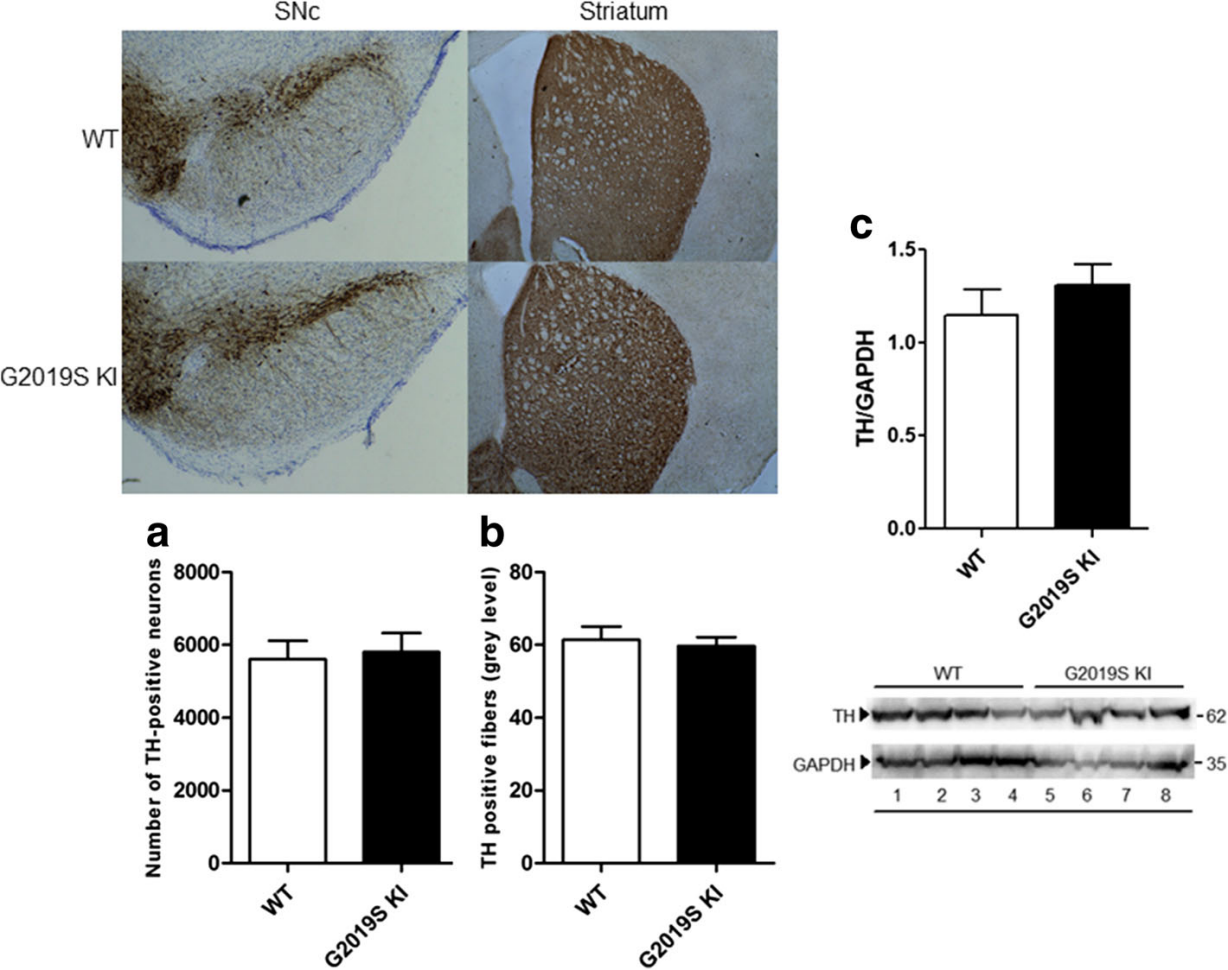
The integrity of nigro-striatal dopaminergic neurons is preserved in G2019S knock-in (KI) mice. Stereological count of nigral DA neurons (**a**) and density of tyrosine hydroxylase (TH) positive striatal nerve terminals (**b**), with representative images, in 12-month-old G2019S KI mice and age-matched WT littermates. Western blotting analysis of striatal TH levels in 12-month-old G2019S KI mice and age-matched WT controls (**c**). Data are expressed as absolute values and are means ± SEM of 8 (**a**-**b**) and 4 (**c**) animals per group

We next investigated the possibility that the G2019S mutation compromises the integrity of nigro-striatal DA neurons (Fig. 2). No differences in nigral DA cell number or density of striatal TH-positive terminals were detected between 12-month-old (Fig. 2a and b, respectively) or 19-month-old (data not shown) G2019S KI and WT mice. Likewise, striatal TH levels were similar between genotypes in 12-month-old animals (Fig. 2c).

### Striatal DA release is preserved in G2019S KI mice

**Fig. 3:**
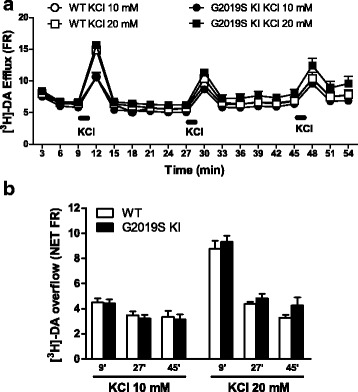
Dopamine (DA) release is preserved in G2019S knock-in (KI) mice. [^3^H]-DA preloaded synaptosomes obtained from the striata of 12-month-old G2019S KI mice and age-matched WT littermates were continuously superfused with Krebs and stimulated with 3 pulses (90 s) of 10 mM or 20 mM K^+^ (18 min apart). DA release has been expressed as fractional release (FR; i.e. tritium efflux expressed as percentage of the tritium content in the filter at the onset of the corresponding collection period; **a**), or NET FR (i.e. K^+^-evoked tritium overflow as percent of the tritium content in the filter at the onset of the corresponding collection period; **b**). Data are means ± SEM of 9 determinations per group

**Fig. 4:**
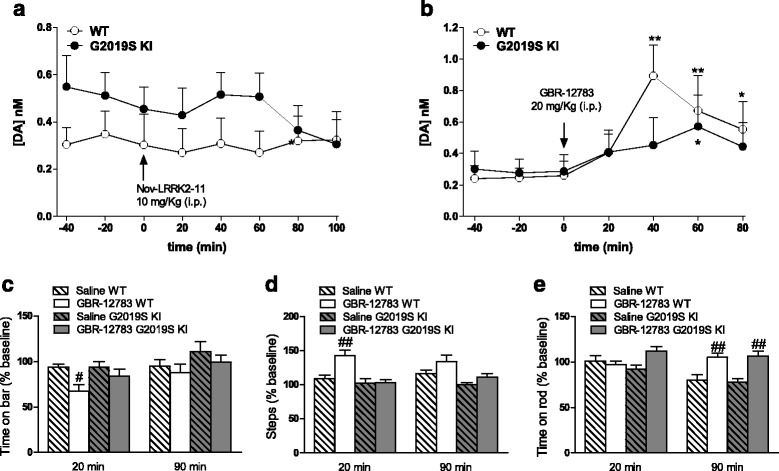
Acute blockade of LRRK2 kinase activity does not affect striatal dopamine (DA) levels whereas acute DAT blockade evoked blunted neurochemical and behavioral responses in G2019S knock-in (KI) mice in vivo. Microdialysis was performed in the dorsolateral striatum of 19-month-old G2019S KI mice and with age-matched wild-type (WT) littermates (WT) (**a**, **b**). Mice were then challenged with the LRRK2 kinase inhibitor Nov-LRRK2-11 (10 mg/kg, i.p.) or the DAT blocker GBR-12783 (20 mg/kg, i.p.). Dialysate levels of DA are expressed as absolute values (nM) and are mean ± SEM of 5 WT and 6 G2019S KI mice (**a**), or 6 WT and 9 G2019S KI mice (**b**). Motor responses of 12-month-old mice to GBR-12783 (6 mg/kg, i.p.) or saline administration (**c**-**e**). Motor activity was assessed using the bar (**c**), drag (**d**) and rotarod (**e**) tests, before (baseline) and after (20 and 90 min) drug administration, and was expressed as percentage of performance at baseline. Data are means ± SEM of 12–17 (WT) or 13–17 (G2019S KI) mice per group. Statistical analysis was performed using one-way RM ANOVA (**a**-**b**) or conventional ANOVA (**c**-**e**) followed by the Newman–Keuls test for multiple comparisons. *p < 0.05, ** *p* < 0.01 significantly different from baseline values; ^#^ *p* < 0.05, ^##^ *p* < 0.01 significantly different from saline

To investigate whether the exocytotic properties of DA terminals were affected by the G2019S mutation (Fig. 3), synaptosomes obtained from the striatum of 12-month-old mice were depolarized with a sequence of three 90-s pulses (18 min away) of 10 mM or 20 mM K^+^ (Fig. 3a). No differences in spontaneous [^3^H]-DA efflux (Fig. 3a) and K^+^-evoked [^3^H]-DA overflow (Fig. 3a,b) were observed between G2019S KI mice and aged-matched WT controls, both after a single or repeated pulses, suggesting that enhanced LRRK2 kinase activity is not associated with changes of striatal DA release. Consistently, in vivo microdialysis revealed no significant differences in dialysate levels of DA and DA metabolites (DOPAC, HVA and 3-MT) between 19-month-old G2019S KI mice and WT littermates (Table 1), although a trend for higher DA and lower metabolites levels in G2019S KI mice was observed. Indeed, significant reductions of HVA/DA and 3-MT/DA ratios in G2019S KI mice were found, the reduction of DOPAC/DA ratio being close to significance (*p* = 0.067; Table 1), suggesting a slower DA metabolism in G2019S KI mice Microdialysis also revealed that the LRRK2 kinase inhibitor Nov-LRRK2-11 (10 mg/kg, i.p.), which normalizes motor performance in G2019S KI mice [43], did not affect striatal DA release in any genotypes (Fig. 4a), suggesting the motor phenotype of G2019S KI mice did not rely on greater DA release.

**Table 1:** Basal dialysate levels (nM) of DA and its metabolites DOPAC, 3-MT and HVA monitored using in vivo microdialysis in the dorsal striatum of 19-month-old G2019S knock-in mice (G2019S KI) and wild-type littermates (WT)

genotype		DA	DOPAC	HVA	3-MT	DOPAC/DA	HVA/DA	3-MT/DA
WT	nM	0.28 ± 0.06	47.31 ± 15.98	82.39 ± 21.44	0.99 ± 0.51	247.63 ± 112.25	242.30 ± 36.65	2.96 ± 0.87
G2019S KI	nM	0.36 ± 0.06	22.12 ± 5.63	54.74 ± 11.63	0.42 ± 0.082	48.65 ± 10.39	114.00 ± 36.50*	1.14 ± 0.24*

*p < 0.05, significantly different from WT The metabolite/DA ratios are also reported. Data are means ± SEM of 11–15 determinations per group and were analyzed using the Student *t*-test, two-tailed for unpaired data

### Age-dependent dysfunction of DAT in G2019S KI mice

Since extracellular DA levels strongly rely on DAT activity, we investigated whether the trend for an increase in extracellular DA levels observed in G2019S KI mice was associated with changes in DAT activity. Microdialysis showed that striatal DA levels were elevated in both genotypes after administration of the DAT blocker GBR-12783 (20 mg/Kg, i.p) (Fig. 4b). However, the response in WT mice was more rapid and larger (maximum ∼ 3-fold over basal) compared to that in G2019S KI mice that was delayed and blunted (∼2-fold over basal) (Fig. 4b).

To confirm dysfunctional DAT activity, we monitored motor performances following GBR-12783 administration. As previously reported [43], G2019S KI mice were more active (*p* < 0.001) in the bar and drag tests (18.29 ± 1.62 s and 13.67 ± 0.47 steps, respectively; *n* = 60) compared to WT littermates (31.57 ± 1.65 s and 9.92 ± 0.39 steps, respectively; *n* = 58). Conversely, rotarod performance was similar in G2019S KI and WT mice (837.58 ± 21.73 and 872.2. ± 31.89 s, respectively). GBR-12783 (6 mg/Kg) reduced the immobility time (Fig. 4c) and increased the stepping activity (Fig. 4d) in WT but not G2019S KI mice, while causing a delayed increase in rotarod performance in both genotypes (Fig. 4e).

**Fig. 5:**
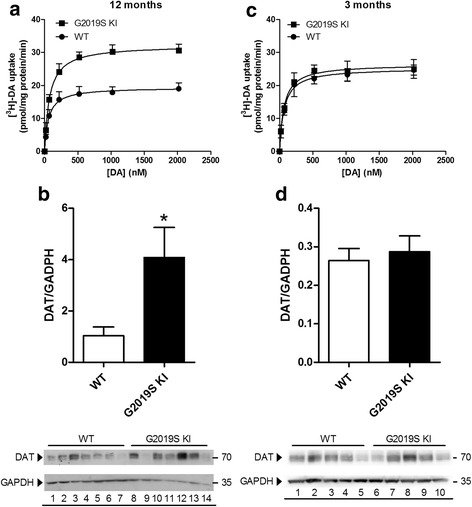
Age-dependent dysfunction of DAT expression and function in G2019S knock-in (KI) mice. Kinetic analysis of [^3^H]-DA uptake in synaptosomes (**a**, **c**), and Western blotting analysis of DAT protein levels (and representative blots) (**b**, **d**) were performed in the striata of 12-month-old (**a**, **b**) and 3-month-old (**c**, **d**) G2019S KI mice in comparison with age-matched WT controls. Values are expressed as mean ± SEM of *n* = 4 (uptake) or *n* = 3 (Western blotting) independent experiments performed in duplicate. Statistical analysis was performed using the Student *t*-test, two-tailed for unpaired data. *p < 0.05, different from WT

We then investigated DAT expression and function in striatal synaptosomes from 12-month-old mice (Fig. 5a, b). Analysis of DA uptake kinetics (Fig. 5a) revealed a significant 63% increase of maximal transport rate (V_max_) in striatal synaptosomes from G2019S KI mice (33.1 ± 1.4 pmol/mg prot/min) with respect to WT mice (20.2 ± 1.1 pmol/mg prot/min; *p* < 0.01), without changes in the DA affinity for the transporter (K_m_ 76.3 ± 8.5 nM vs 67.9 ± 9.0 nM in G2019S KI and WT mice, respectively). Consistent with higher V_max_, Western blot analysis showed that DAT protein levels were ~4-fold higher in G2019S KI than WT mice (Fig. 5b). To investigate whether these changes were age-dependent, experiments were replicated in younger animals (Fig. 5c,d). No differences were observed in [^3^H]-DA uptake kinetics between 3-month-old G2019S KI mice (K_m_ 66.2 ± 10.1 nM, V_max_ 26.5 ± 1.7 nM) and age-matched WT controls (K_m_ 70.5 ± 10.6 nM, V_max_ 25.3 ± 0.6 nM) (Fig. 5c). Likewise, protein levels were similar between genotypes at this age (Fig. 5d).

### Age-dependent dysfunction of VMAT2 in G2019S KI mice

**Fig. 6:**
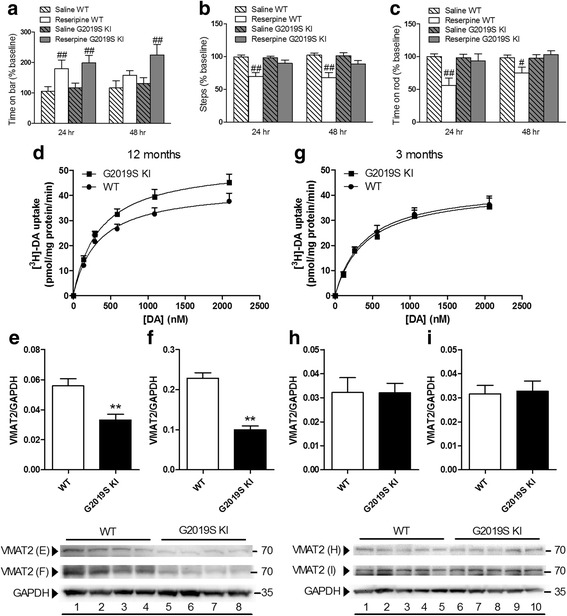
Age-dependent dysfunction of VMAT2 expression and function in G2019S knock-in (KI) mice. Motor activity in 12-month-old G2019S KI mice and wild-type (WT) littermates treated with reserpine (1 mg/kg, i.p.) or saline, and challenged in the bar (**a**), drag (**b**) and rotarod (**c**) tests, before (baseline) and after (24 and 48 h) drug administration. Motor performance was expressed as percentage of performance at baseline. Data are means ± SEM of *n* = 14–15 mice per group and were analyzed using conventional ANOVA followed by the Newman–Keuls test for multiple comparisons. ^#^ *p* < 0.05, ^##^ *p* < 0.01 significantly different from saline. Kinetic analysis of [^3^H]-DA uptake in whole-brain vesicles and Western blotting of VMAT2 levels in the striata from 12-month-old (d-f) or 3-month old (**g**- **i**) G2019S KI mice and WT littermates. In Western blotting, two different anti-VMAT2 antibodies were used, one commercially available (**e**, **h**; Sigma) and another developed by Miller lab (**f**, **i**) (see Methods). Data are expressed as mean ± SEM of 4 mice (**d**- **f**), 3 mice (**g**) or 5 mice (**h**, **i**) per group, performed in duplicate. Statistical analysis was performed by the Student *t*-test, two-tailed for unpaired data. **p < 0.01, different from WT

Since the DAT/VMAT2 ratio is a vulnerability factor in DA neurons [56], we next investigated whether VMAT2 was also dysfunctional in G2019S KI mice (Fig. 6). First, the VMAT2 blocker reserpine was administered (1 mg/Kg, i.p.) to 12-month-old mice (Fig. 6a-c). G2019S KI and WT mice showed similar increases of immobility time 24 h after reserpine administration, although G2019S KI mice were also affected at 48 h (Fig. 6a). Conversely, reserpine reduced stepping activity (Fig. 6b) and rotarod performance (Fig. 6c) selectively in WT mice, both at 24 h and 48 h after administration. Higher reserpine doses (2 mg/Kg), however, caused similar motor impairments in both genotypes (data not shown). We then measured VMAT2 uptake activity in a preparation of whole-brain synaptic vesicles (Fig. 6d). VMAT2 affinity for DA (K_m_) was similar between 12-month-old G2019S KI mice and WT controls (356.3 ± 25.2 vs 333.6 ± 31.0 nM, respectively), although V_max_ was significantly higher in G2019S KI mice (52.7 ± 2.4 vs 43.2 ± 2.2 nM, respectively; *p* < 0.05). Striatal VMAT2 protein levels were then analyzed, comparing a commercially available (Fig. 6e) with an in-house validated [15] antibody (Fig. 6f). Both antibodies revealed a ~50% reduction of VMAT2 levels in G2091S KI mice. Finally, VMAT2 activity and protein levels were measured in 3-month-old mice (Fig. 6g-i). As for DAT, no differences between genotypes were observed at this age.

### Age-dependent increase of pSer129 α-syn in G2019S KI mice

**Fig. 7:**
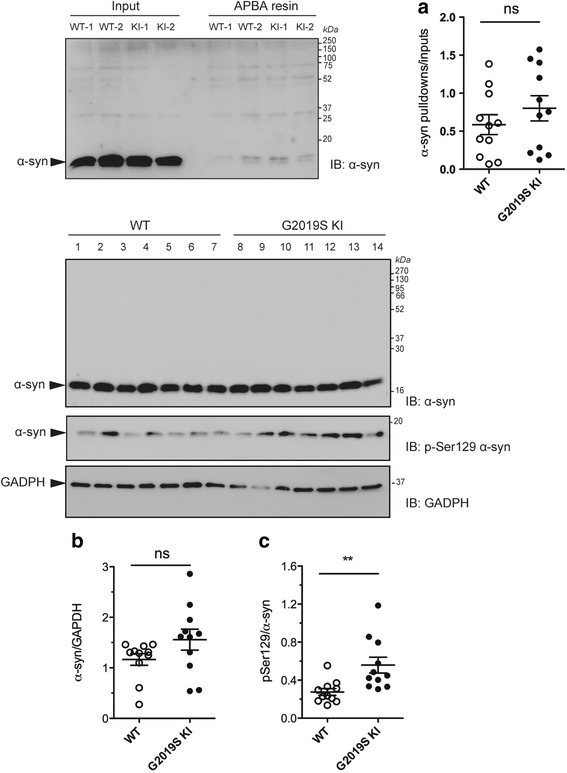
DOPAL-modified α-synuclein (α-syn) levels are unchanged whereas Ser129-phosphorylated α-synuclein (pSer129 α-syn) levels are elevated in G2019K knock-in (KI) mice. Relative quantification and representative blots of DOPAL-bound α-syn pull-down with aminophenylboronic acid (APBA) resin of striata from 12-month-old G2019S KI mice and age-matched WT controls (**a**). In the same preparation, pSer129 α-syn levels (**c**) were quantified relatively to α-syn levels (**b**). Data are expressed as mean ± SEM of *n* = 11 mice per group. Statistical analysis was performed by the Student *t*-test, two tailed for unpaired data. **p < 0.01 different from WT

**Fig. 8:**
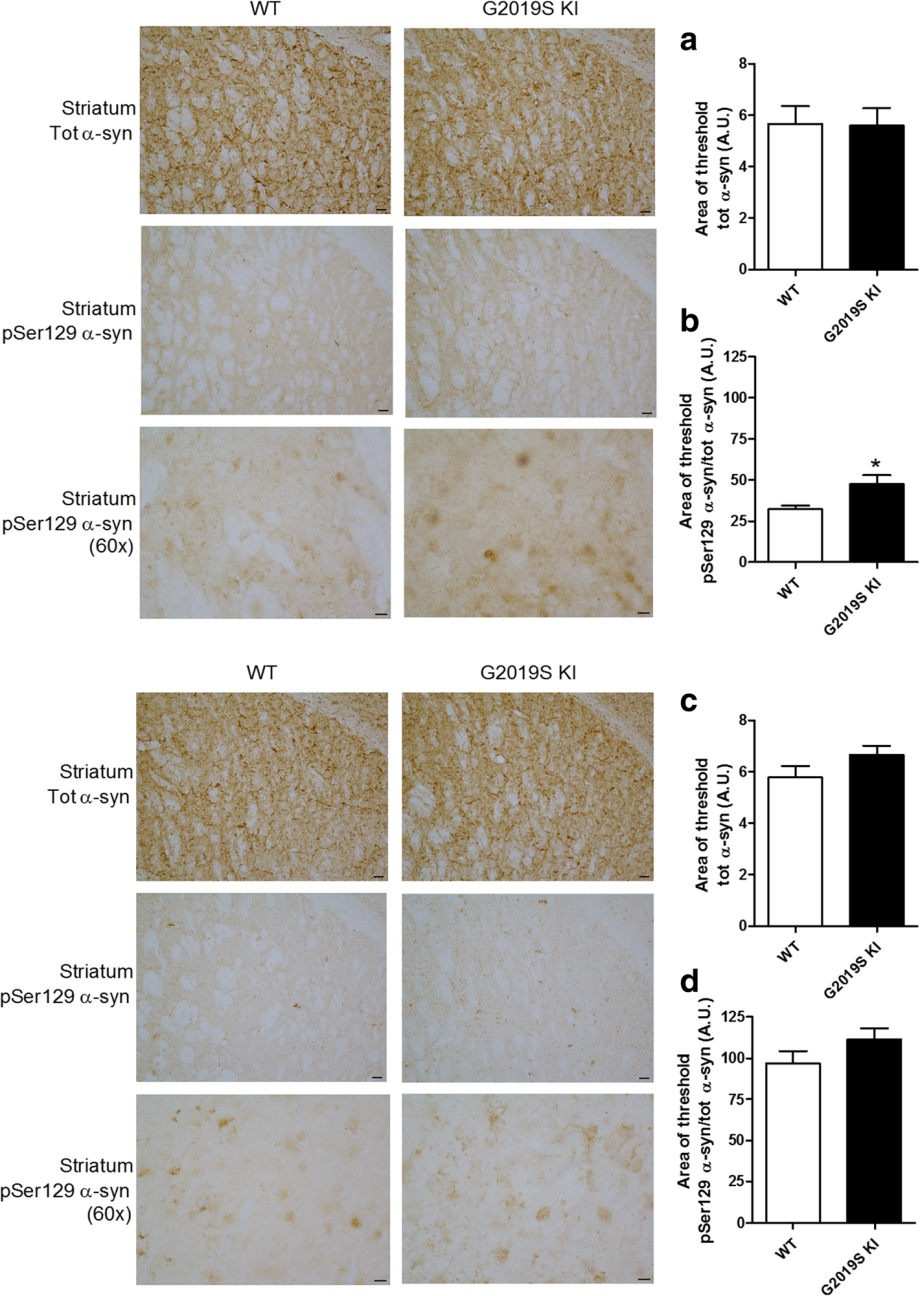
Age-dependent overload of Serine129-phosphorylated α-synuclein (pSer129 α-syn) in G2019S knock-in (KI) mice. Representative microphotographs and relative quantifications of α-syn and pSer129 α-syn immunostaining in the striatum of 12-month-old (**a**, **b**) and 3-month-old (**c**, **d**) G2019S KI mice and WT controls. Data are expressed as mean ± SEM of 8 (**a**, **b**) or 6 (**c**, **d**) mice per group. Statistical analysis was performed by the Student *t*-test, two tailed for unpaired data. *p < 0.05 different from WT

An increase of DAT activity [50] can lead to an increased cytosolic DA levels and buildup of byproducts of DA metabolism which are toxic for the cell. Among these, DOPAL [8, 52, 60], which is known to covalently bind to α-syn at lysine residues [21, 89]. We therefore measured DOPAL-bound α-syn levels in the striatum of 12-month-old G2019S KI mice compared to WT controls (Fig. 7a). No significant difference between genotypes was found. We then investigated pSer129 α-syn levels (Fig. 7b, c), since this posttranslational modification of α-syn is thought to influence α-syn aggregation and is highly represented in intracellular inclusions and Lewy Bodies [1, 24, 69]. Total endogenous α-syn levels were not different between genotypes (Fig. 7b) whereas pSer129 α-syn levels were ~2-fold higher in the striatum of G2019S KI mice (Fig. 7c). To study the localization of α-syn and pSer129 α-syn, immunohistochemistry was employed in striatal slices from 12-month-old mice (Fig. 8). α-syn and pSer129 α-syn inclusions were revealed in striatal neurons of both genotypes, seemingly at the cell body level. Endogenous α-syn levels did not differ between genotypes (Fig. 8a), while pSer129 α-syn inclusions were significantly higher in the striatum of G2019S KI mice compared to WT controls (Fig. 8b). We next confirmed that such increase was age-dependent, since no difference in striatal α-syn or pSer129 α-syn levels was observed between 3-month-old G2019S KI mice and WT controls (Fig. 8c, d).

## Discussion

We previously reported that G2019S KI mice have a hyperkinetic phenotype relying on elevated kinase activity [43]. In this follow-up, we show that 12-month-old G2019S KI and WT mice bear similar numbers of nigral DA neurons and striatal DA terminals, in keeping with previous studies in the same genotype [29, 92] or in BAC G2019S overexpressing mice [40, 53] and rats [37, 74, 93], as well as similar extracellular DA levels and depolarization-evoked striatal DA release, in line with that found in 22-month old R1441G KI mice [82]. Since the LRRK2 kinase inhibitor Nov-LRRK2-11 [43] also failed to affect striatal DA release in vivo in any genotypes, we conclude that the nigro-striatal DA system is morphologically intact and the exocytotic properties of DA neurons are functionally preserved in G2019S LRRK2 carriers.

The lack of changes of striatal DA release is at striking variance with the 60% reduction in basal striatal extracellular DA levels reported, in the absence of motor phenotype change, in 12-month-old G2019S KI mice [92]. We cannot easily explain this difference since both strains of G2019S KI mice are backcrossed on C57BL and bear similar kinase-enhancing mutations on exon 41 [29, 92]. Indeed, kinase activity appears to be elevated in both strains, as evaluated by in vitro kinase assays on synthetic substrates [29, 92]. We confirmed this finding in vivo, showing that, in good agreement with previous work on brain lysates of BAC G2019S mice [72], pSer1292 levels were ~8-fold higher in the striatum of G2019S KI mice compared to WT controls. pSer1292 appears a more reliable marker of kinase activity with respect to ATP γ-phosphate incorporation measured in in vitro assays. Indeed, Ser1292 LRRK2 is an autophosphorylation site, and pSer1292 levels correlate with in vivo kinase activity [72]. pSer1292 levels are a more reliable readout of in vivo LRRK2 kinase activity even compared to pSer935 levels, since LRRK2 is phosphorylated at Ser935 by other kinases [14, 20, 67]. The discrepancies between these two strains of G2019S KI mice might be explained by quantitative differences in kinase activity along with inter-individual genomic variability, motor tests used, or environmental conditions.

Despite the lack of changes of DA release, the levels and functions of proteins involved in DA synaptic load (DAT) and vesicular storage (VMAT2) were altered in 12-month-old G2019S KI mice. Strikingly, these changes were age-dependent, since they were not observed in 3-month-old animals, indicating these changes are elements of an orchestrated, progressive response relying on the interaction between a genetic factor (the G2019S mutation) and aging, i.e. the main risk factor in PD.

DAT was upregulated in G2019S KI mice, which might represent a vulnerability factors for DA neurons [56]. Indeed, DAT overexpression has been associated with an increase of oxidative stress and neuronal degeneration [50] likely because cytosolic DA accumulation causes the buildup of reactive oxygen species and quinones, generated by DA autoxidation [25, 77, 79]. Moreover, cytosolic DA is metabolized by monoamine oxidase A to DOPAL, which causes synaptic dysfunction and terminal loss acting via different mechanisms, including cross-linking with α-syn [9]. Finally, environmental toxins causing PD, such as the toxic metabolite of MPTP, MPP^+^, are taken up by DA neurons through DAT. In fact, the greater susceptibility of BAC hG2019S overexpressing mice to the parkinsonian toxin MPTP can be explained by DAT upregulation [32].

Quite paradoxically, the increase of DAT activity was associated with a blunted neurochemical and behavioral response to GBR-12783. This is consistent with microdialysis works reporting a 35–50% reduction of nomifensine-induced DA release in the striatum of KI mice constitutively expressing R1441G LRRK2 [39] or temporally expressing G2019S LRRK2 [93]. Previous studies in cells have shown that DAT expression levels inversely correlate with the potency of DAT blockers [12], a phenomenon also observed for the serotonin transporter [65]. Membrane DAT is in equilibrium between oligomeric and monomeric forms, and it has been hypothesized that higher DAT expression leads to higher DAT oligomerization, and DAT oligomers have lower affinity for DAT blockers with respect to monomers [38].

In line with that found in G2019S overexpressing mice [41], G2019S KI mice showed reduced striatal VMAT2 levels. This reduction was robust and consistent with the two different antibodies, one of which validated in VMAT2^+/-^ mice [15]. Reduction of VMAT2 is observed also in PD patients [55] and is pathogenic in PD. In fact, filling synaptic vesicles via VMAT2 is a way to keep cytosolic DA levels in a nontoxic range; accordingly, VMAT2 deletion induces neurodegeneration [80] whereas VMAT2 overexpression protects DA neurons [10, 42]. However, despite VMAT2 reduction further enhanced the already higher DAT/VMAT2 ratio in nigro-striatal DA neurons, thus increasing their vulnerability [56], G2019S KI mice did not show overt neurodegeneration (up to 19-months at least) or even significantly enhanced levels of DOPAL-bound α-syn, a marker of DA cytotoxicity. This questions the physiological meaning of the 50% reduction of VMAT2 observed in the striatal homogenate of G2019S KI mice. Indeed, contrary to that expected from the Western blot data, an increase in tetrabenazine-sensitive vesicular DA uptake was measured in G2019S KI mice in vitro. Although we cannot rule out the possibility that such increase is compensatory in nature, the possibility that this discrepancy relies on technical reasons should be considered. In fact, the reduction of VMAT2 levels measured in striatal homogenate might not faithfully reflect a reduction of active VMAT2 expressed on mature, release-prone synaptic vesicles. In fact, VMAT2 levels measured by Western blot encompass also VMAT2 contained in immature secretory vesicles trafficking from the soma to presynaptic vesicle membrane, or recycling from the plasma membrane [30]. Interestingly, Sonsalla and collaborators [30] proved a disparity between tetrabenazine binding measured in striatal homogenate and striatal synaptic vesicles at 24 h after MPTP, showing that, under certain conditions, tetrabenazine binding measured in striatal homogenate may not be representative of vesicular VMAT2. On the other hand, the major limitation of a whole-brain preparation of synaptic vesicles is heterogeneity. VMAT2 is present not only in striatal dopaminergic terminals but also in noradrenergic, serotoninergic and histaminergic terminals in striatal and extrastriatal areas. Since there is no possibility to dissect out the contribution of the different populations of VMAT2-positive synaptic vesicles in this whole-brain preparation, we cannot prove that the observed increase of vesicle uptake is really due to VMAT2 expressed on striatal vesicles, or is the net result of all changes of VMAT2 activity in different nerve terminals and brain areas.

Nonetheless, in favor of the hypothesis that vesicular DA uptake might be increased rather than reduced in G2019S KI mice, G2019S KI mice were relatively more resistant than WT controls to the hypolocomotive action of 1 mg/Kg reserpine in vivo, which is opposite from that expected from DA depleted vesicles. We can speculate that the greater resistance to reserpine might be due to a greater competition for VMAT2 of reserpine and cytosolic DA (the increase in DAT activity and the reduced DA turnover might overwhelm the buffering capacity of VMAT2, thus causing an increase in cytosolic DA). Alternatively, we might speculate that synaptic vesicles in G2019S KI mice are more enriched in DA, although only a trend to an increase in extracellular DA levels or in the K^+^-induced DA release was observed in the G2019S LRRK2 carriers.

It is therefore plausible that VMAT2 uptake elevation compensates for the loss of VMAT2 protein and protects from cytosolic DA toxicity, even in the presence of upregulated DAT. Whether this adaptive change will be effective throughout the life-span of G2019S KI mice is unknown, since we have investigated G2019S KI mice up to 19 months. However, it is also possible that other compensatory mechanisms will come into play to preserve DA homeostasis and DA neuron integrity.

In this respect, one important finding of the present study is that pSer129 α-syn levels are elevated in the striatum of G2019S KI mice. Since this was not paralleled by an elevation of total α-syn levels, we concluded that G2019S LRRK2 facilitates this posttranslational modification of α-syn. This is in line with a recent study showing that the formation of pSer129 α-syn-positive inclusions in nigral DA neurons in response to intranigral α-syn fibrils injection is accelerated in BAC hG2019S rats [86]. pSer129 α-syn [24] is the predominant form of syn in Lewy bodies [1], and for this reason it has been hypothesized to favor α-syn aggregation, thus contributing to PD [57]. However, the role of pSer129 α-syn phosphorylation in α-syn toxicity in vivo is still under debate [57, 81]. In fact, from the published literature it appears that depending on which kinase is involved in Ser129 α-syn phosphorylation, either neurotoxicity (G protein receptor kinases) [11, 71] or neuroprotection (Polo-like kinase 2) [58] can ensue. Moreover, LRRK2, and more intensely G2019S LRRK2, can directly Ser129-phosphorylate α-syn in vitro [63]. Pinning down the pathway underlying Ser129 α-syn phosphorylation might help understand whether this modification is protective or pathogenic for DA neurons.

## Conclusion

We previously reported that G2019S KI mice have enhanced motor performance starting at 6 months of age [43]. We now show that this behavior is not sustained by enhanced DA release, suggesting that other mechanisms, such amplified postsynaptic D1 receptor signalling [54, 61] or glutamate release [3], might contribute. Of note, this study reveals for the first time that G2019S KI mice progressively develop (between 3 and 12 months) dysfunctions of plasma membrane and vesicular DA transporters, along with an overload of pSer129 α-syn inclusions in striatum. These adaptive changes were not associated with overt nigro-striatal DA degeneration or changes of striatal DA release, indicating DA homeostasis is preserved, at least up to 19-months. Nonetheless, they might represent vulnerability factors to DA neurons. A more stringent analysis of the time-course of these changes might help elucidate how this response of DA terminals is orchestrated, and how these factors relate to each other and, ultimately, to G2019S LRRK2. In fact, there is no evidence that G2019S LRRK2 can directly affect DAT or VMAT2 trafficking, although this possibility is worth investigating due to the role of LRRK2 in endosome and autophagosome pathways [68]. Nonetheless, G2019S LRRK2 could do so indirectly, through pSer129 α-syn. Indeed, α-syn stimulates DAT activity [23, 36] and G2019S LRRK2 has been shown to increase this property by phosphorylating α-syn at Ser129 [27]. Preliminary evidence that α-syn controls VMAT2 activity has also been collected in cells where α-syn knockdown causes an increase and A53T α-syn overexpression a reduction [22, 44]: nonetheless, the role of the pSer129 remains unknown. Whatever the mechanistic interactions between these players are, the gradual development of this response offers a wide time-window for a pharmacological intervention (e.g. with LRRK2 inhibitors) that could establish the role of the kinase vs non kinase activities of LRRK2.

In conclusion, G2019S KI mice might represent a presymptomatic model of PD, a valuable tool to verify a “multi-hit” hypothesis of PD [78], where genetic variables (G2019S LRRK2), an established risk factor (aging), and internal (e.g. DA, α-syn) or environmental (e.g. MPTP) factors interact to shape the emergence of the parkinsonian phenotype.
